# Sex, age and generation effects on genome-wide linkage analysis of gene expression in transformed lymphoblasts

**DOI:** 10.1186/1753-6561-1-s1-s92

**Published:** 2007-12-18

**Authors:** Jagadish Rangrej, Joseph Beyene, Pingzhao Hu, Andrew D Paterson

**Affiliations:** 1 Programs in Genetics and Genome Biology, The Hospital for Sick Children, 101 College Street, TMDT East Tower, Toronto, Ontario M5G 1X8 Canada; 2 Child Health Evaluative Sciences, The Hospital for Sick Children, 123 Edward Street, Toronto, Ontario, M5G 1X8 Canada; 3 Department of Public Health Sciences, University of Toronto, 155 College Street, Toronto, Ontario, M5T 3M7 Canada

## Abstract

**Background:**

Many traits differ by age and sex in humans, but genetic analysis of gene expression has typically not included them in the analysis.

**Methods:**

We used Genetic Analysis Workshop 15 Problem 1 data to determine whether gene expression in lymphoblasts showed differences by age and/or sex using generalized estimating equations (GEE). We performed quantitative trait linkage analysis of these genes including age and sex as covariates to determine whether the linkage results changed when they were included as covariates. Because the families included in the study all contain three generations, we also determined what effect inclusion of generation in the model had on the age effects.

**Results:**

When controlling the false-discovery rate at 1%, using GEE we identified 30 transcripts that showed significant differences in expression by sex, while 1950 transcripts showed differences in expression associated with age. When subjected to linkage analysis, there were 37 linkages that disappeared, while 17 appeared when sex was included as a covariate. All these genes were, as expected, on the sex chromosomes. In contrast, when age was included in the linkage analysis, 462 linkage signals were no longer significant, while 223 became significant. When generation was included in the model with age, all but 6 of the GEE age effects were no longer significant. However, there were minimal changes in the linkage results.

**Conclusion:**

The effect of age on linkage analyses was apparent for the expression of many genes, which appear to be mostly due to differences between the generations.

## Background

Many traits in humans differ by sex and age, but analyses of gene expression typically do not include them as covariates [1]. It has been shown in simulation studies that incorporating appropriate covariates in linkage analysis improves power without compromising type I error [2]. We were interested to determine if there are loci that influence gene expression whose detection is conditional on inclusion or exclusion of age and/or sex from the analysis. In addition, since the data were from three-generation families, we determined to what extent the age effects are accounted for by generation effects.

## Methods

### Association between age and sex, and gene expression

We used the 3554 transcripts that were reported to show greater variation between than within 94 grandparents from CEPH (Centre d'Etude du Polymorphisme Humain) pedigrees [1]. Age data were obtained from ; they were not available for any member of pedigree 1454 and three individuals from three other families. We used generalized estimating equations (GEE) [3] in the computer program R to test whether the expressions of each gene differed by age and sex using family as a clustering variable and using an exchangeable correlation structure. In addition, indicators for generation were also added to the model. We calculated *q*-values to estimate the false-discovery rate for covariate effects [4].

### Expression quantitative trait linkage analysis

Expression quantitative trait linkage (eQTL) analysis was performed using MERLIN-REGRESS v 1.0.1 with a bug fix for missing covariates [5,6]. Mendelian inconsistencies between grandparents and children were removed. Marker allele frequencies for 2871 single-nucleotide polymorphisms (SNPs) were estimated from the data and single-point linkage analysis was used (10,203,534 tests). Variance-components linkage analysis, using MERLIN, was used to analyze the X chromosome because MERLIN-REGRESS cannot analyze X chromosome data. We present results using the following criteria for considering linkage results to be different between the analysis with and without covariates: linkage results with a LOD score >3 and either a 3 LOD unit increase or decrease in linkage when sex or age was included as a covariate. Linkage analysis including age and both age and generation were used to determine what effect including generation had on linkage results.

## Results

### Association between age, sex, generation and gene expression

**Figure 1:**
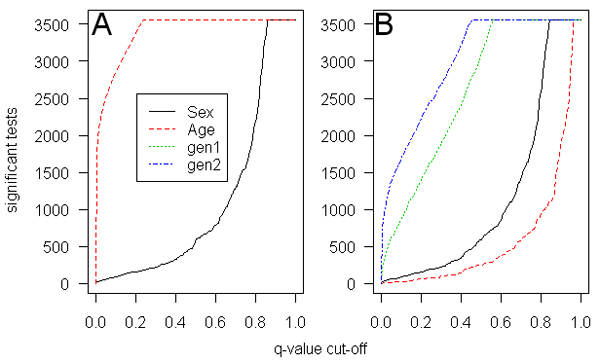
**Distribution of the number of gene expressions that are significant in the GEE model for the range of *q*-values**. A, Covariates are sex and age; B, sex, age, and grandparental (gen1) and parental (gen2) generation.

Descriptive information regarding age and sex are provided in Table 1. From the GEE analysis of gene expression data, Figure 1A shows the distribution of the number of significant tests if different *q*-value thresholds are used for the models with age, sex, and generation. After adjustment for familial correlation there were 30 genes that showed significant differences by sex, compared to 1950 genes that were significantly associated with age (an FDR threshold of 0.01 was used). The respective *p*-values for test of significance were 0.000097 for sex and 0.023 for age. When generation was included in the model (Fig. 1B) only 6 of the age effects remained significant, while generation effects were significant for 277 and 862 genes, for the indicators for the grandparental (g1) and parental (g2) generations respectively (the number of genes with sex effects (29) remained similar). The *p*-values for this model were: sex = 8.1 × 10^-5^, age = 1.6 × 10^-5^, g1 = 0.0014, and g2 = 0.0053.

**Table 1:** Descriptive information about age and sex

			^a^Age		
			
Sex	*N*	*N *missing age	Grandparent	Parent	Child
Male	99	8	73 ± 9 (61–92)	46 ± 7 (39–66)	17 ± 7 (5–34)
Female	95	9	70 ± 7 (61–85)	46 ± 5 (39–59)	18 ± 8 (5–37)

^a^Ages are given as mean ± SD (range).

### Expression quantitative trait linkage analysis

**Figure 2:**
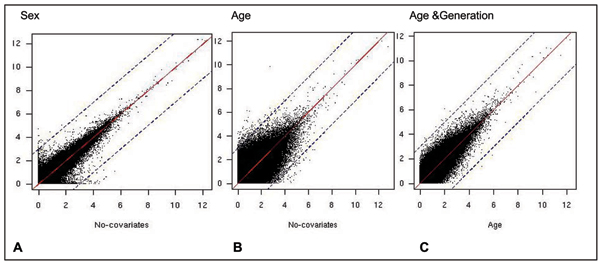
**Distribution of LOD scores when covariates are included in linkage analysis**. Sex (A) or age (B) were included as covariates and are plotted on vertical axis, compared to no covariates on the horizontal axis. C, On the vertical axis age and generation were included as covariates, compared to age as a covariate on the horizontal axis. The solid line indicates symmetry, the dashed lines are at ±3 LOD scores from symmetry.

Figure 2 shows the LOD scores with and without sex and age, respectively. Note the greater change in LOD scores when age (Fig. 2B) was included as a covariate than when sex (Fig. 2A) was included. Specifically, 37 linkage signals disappear and 17 appear when sex was included. As expected, all of those traits with linkage results that changed when sex was included map to the sex chromosomes, and the loci showing changes in linkage were on the autosomes (Table 2). Of particular interest were the four genes that are on the X chromosome and for which linkage signals appear on the autosomes when sex is included. This suggests that autosomal loci influence the expression of some genes that escape X-inactivation. For the age analysis, 462 significant linkage results disappeared while 223 appeared when age was included in the analysis. Table 3 provides a list of the 10 top SNPs and traits that show evidence for linkage when age is either included or excluded as a covariate. When linkage analysis was performed with both age and generation as covariates, the change in the linkage results (Fig. 2C) was not as marked as when only age was included as a covariate (Fig. 2B). According to our criteria, only four linkage results disappeared when generation was added to age, and none appeared.

**Table 2:** Marked changes in linkage when sex was included as a covariate

SNP^a^		Trait		LOD scores		
		
Rs no.	Location Chr:Mb	Gene	Location Chr:Mb	Without sex	With sex	Change when sex included
rs1515504	6:11.1	*SMCY*	Y:20.3	0.00	4.68	4.68
rs1885567	20:16.6	*DDX3Y*	Y:13.5	0.02	4.12	4.11
rs1861606	12:22.3	*SMC1L1*	X:53.4	0.83	4.43	3.60
rs239345	16:23.3	*UTY*	Y:14.0	0.11	3.69	3.57
rs216896	12:6.0	*STS*	X:7.2	0.17	3.75	3.57
rs1959305	15:26.1	*EIF1AX*	X:20.1	0.06	3.63	3.57
rs540224	3:39.0	*EIF1AY*	Y:21.2	0.03	3.58	3.55
rs411602	18:6.1	*EIF1AY*	Y:21.2	0.00	3.49	3.49
rs2027871	20:42.6	*DDX3Y*	Y:13.5	0.00	3.43	3.43
rs1933121	14:37.4	*EIF1AY*	Y:21.2	0.29	3.66	3.37
						
rs1884910	20:54.5	*XIST*	X:13.2	5.55	1.03	-4.52
rs739495	19:40.4	*USP9Y*	Y:13.4	4.76	0.36	-4.41
rs1892687	21:35.2	*SMCY*	Y:20.3	4.40	0.00	-4.40
rs1979797	7:32.6	*XIST*	X:13.2	4.82	0.43	-4.39
rs983740	10:20.8	*DDX3Y*	Y:13.5	4.95	0.60	-4.35
rs1055403	7:30.7	*EIF1AY*	Y:21.2	4.46	0.15	-4.30
rs715257	10:13.2	*DDX3Y*	Y:13.5	4.16	0.00	-4.16
rs983740	10:20.8	*EIF1AY*	Y:21.2	4.25	0.16	-4.09
rs715257	10:13.2	*EIF1AY*	Y:21.2	4.07	0.00	-4.07
rs1364161	17:2.1	*DDX3Y*	Y:13.5	4.14	0.09	-4.05

^a^The 10 top SNPs and traits for which there is evidence for linkage when sex either included or excluded as a covariate are shown as examples.

**Table 3:** Marked changes in linkage when age was included as a covariate

SNP^a^		Trait		LOD scores		
		
Rs no.	Location Chr:Mb	Gene	Location Chr:Mb	Without age	With age	Change when age included
rs1505695	02:06.9	*ITGB1BP1*	02:09.5	3.36	9.82	6.47
rs756019	15:21.2	*CREM*	10:35.5	0.98	6.14	5.16
rs1749715	15:30.3	*SCD*	11:42.1	0.92	6.07	5.15
rs1957538	15:21.2	*CREM*	10:35.5	0.05	5.02	4.97
rs753151	08:48.7	*LIF*	22:29.0	0.77	5.53	4.76
rs1499511	11:36.0	*RASA1*	06:26.7	0.55	5.29	4.74
rs1264898	02:51.8	*SNX5*	20:17.9	0.20	4.82	4.62
rs1503230	03:08.0	*LRAP*	06:36.3	0.22	4.67	4.45
rs1417594	15:34.4	*VPS16*	20:02.8	0.33	4.75	4.42
rs1858799	16:23.0	*ZNF277*	08:51.7	0.75	5.16	4.41
						
rs1005989	03:08.9	*DGUOK*	03:14.0	6.40	1.04	-5.36
rs1986778	12:26.9	*TNFAIP8*	06:58.7	6.02	0.74	-5.28
rs1862802	16:57.0	*TLR1*	04:38.5	5.51	0.26	-5.26
rs1333798	14:27.5	*RECQL4*	10:25.7	6.10	1.06	-5.03
rs954779	09:36.4	*CDC25C*	07:17.7	5.23	0.22	-5.00
rs1341446	04:59.6	*PIK3C3*	18:37.9	7.61	2.71	-4.89
rs1935886	03:29.8	*CHI3L2*	02:51.6	11.64	6.78	-4.86
rs1432285	04:14.0	*HNRPR*	01:23.5	5.56	0.74	-4.82
rs1459359	15:27.7	*C3orf4*	04:39.7	5.10	0.28	-4.82
rs744531	03:51.7	*SLC25A32*	09:44.5	5.78	1.00	-4.78

^a^The 10 top SNPs and traits for which there is evidence for linkage when age is either included or excluded as a covariate are shown as examples.

## Discussion

We found that the majority of genes show significant differences in expression by age, while only a subset show significant sex differences. There were more linkage signals that were no longer significant when sex or age were included as covariates than appeared as a consequence of inclusion of these covariates. When generation was also included in the linkage analysis with age, few linkage results changed. Limitations of our analysis include the fact that age data were missing for all individuals in one family and for three individuals in other families.

In addition, we took a blanket approach to all traits because performing detailed diagnostics for numerous traits is not straightforward. To investigate the potential risks of this approach, we examined the trait distributions for the linkage results that changed dramatically when either sex or age was included as covariate (Tables 2 and 3). As expected, many of the traits that had marked sex effects on linkage results were bimodally distributed, which may result in false-positive linkage results [7]. Interestingly, when we repeated linkage analyses for loci where age and sex had marked effects using the variance-components methodology (as opposed to MERLIN-REGRESS), there was little difference between inclusion and exclusion of the covariate, raising concern about the validity of the regression results.

Morley et al. [1] selected genes for linkage analysis based on greater variance between, compared to within, individuals. They performed this analysis on the grandparents: of those available in this data set, the mean age was 72 years (SD = 8, range = 61–92). However, the grandparents were extracted from linkage analysis. Only the traits of children, whose mean age was 18 years (SD = 8, ranges = 3–37) were used for the linkage analysis. Reasons for this may be related to limitation of available methods or a decision to attempt to reduce age effects. However, if the variance is not the same for grandparents and children, then such an approach may results in genes that are falsely included or excluded from the genetic linkage analysis. Furthermore, although in our analysis we used age, the age effects are mostly removed when generation was included. In such a situation age will be highly correlated with birth order within a sibship and therefore we cannot exclude that this has resulted in confounding.

We used a simple exchangeable covariance structure in our GEE analyses. This takes into account the family dependence to some extent but may not be the most appropriate covariance structure for the data. It would be interesting to investigate other covariance structures and assess the impact that they have on the overall findings.

## Conclusion

Age, and to a lesser degree sex, influence gene expression in transformed B lymphocytes. Although including sex as a covariate did not result in many changes in the linkage results, when age was included the results changed more markedly-specifically there were fewer significant linkage results when age was included as a covariate. It appears that many of these age effects can be accounted for by generational differences in gene expression. Inclusion of covariates in quantitative trait linkage analysis may improve power and reduce false positives.

## Competing interests

The author(s) declare that they have no competing interests.
